# Harris JR, Korolchuk VI, eds. Biochemistry and Cell Biology of Aging: Part II Clinical Science: Subcellular Biochemistry; Volume 91; Springer Nature Singapore Pte Ltd 2019;499 pages; ISBN 978-981-13-3680-5; ISBN 978-981-13-3681-2 (eBook)

**DOI:** 10.3325/cmj.2020.61.208

**Published:** 2020-04

**Authors:** Antonela Blažeković, Fran Borovečki

**Field of Medicine:** Science of aging; biochemistry of aging

**Audience:** This book is intended for biomedical science students, medical students, postgraduate researchers, and clinicians interested in all aspects of aging.

**Purpose:** To provide an in-depth survey of numerous aspects within the field of aging research, with the emphasis on clinical sciences.

**Content:** The textbook is divided into 17 clinical science chapters standing firmly on its own merit. Each chapter is written by eminent clinical scientists, with the aim of covering the field in an all-encompassing manner.

The book begins with a chapter dealing with the factors involved in the earliest stages of growth and development and their impact on the processes that occur during aging. The chapter, named Poor Early Growth and Age-Associated Disease, introduces major concepts and discusses the correlation of suboptimal maternal and early postnatal environments with age-associated diseases. The chapter also deals with the progress in the development of safe and effective intervention strategies that could ultimately target the “programmed” individuals who are presumed to be at risk for aging-related disorders.

The next chapters discuss how age-associated changes illicit various disorders, namely chronic lung disease, neurodegenerative diseases, osteoarthritis, Down syndrome, eye diseases, osteoporosis, and soft tissue disorders. The book also describes distinct diseases of accelerated aging, with a profound insight into Hutchinson-Gilford progeria syndrome. The diseases are addressed primarily from a clinical perspective, with a special focus on disease pathophysiology. These chapters also describe the mechanisms by which aging contributes to disease development. A particular emphasis is placed on the description of the main neuropathological features of the most common age-associated neurodegenerative diseases. Neurological changes are described in detail in the context of “pathological” aging but also of “normal” or “healthy” aging.

The book also describes how aging affects different functional systems and pathways, such as the immune system, vestibular system, signal transduction, and neurovascular system. Given the fact that each chapter was authored by different experts, each of them is written in a different style. Some are almost completely written from the clinical perspective (ie, Skin Changes during Aging), while some topics are presented at a highly detailed molecular level (ie, Signal Transduction, Aging and Disease), addressing subcellular events related to aging.

Furthermore, two chapters are devoted to the prevention of age-associated diseases and describe potential cellular and biochemical mechanisms underlying exercise and physical activity, as well as address health benefits of anti-aging drugs.

**Highlights:** The book addresses particular problems from a clinical perspective. However, it also intricately connects molecular aspects with genetic and epigenetic changes, thus enriching the understanding of the underlying processes involved in aging-related disorders.

**Limitations:** The chapters are self-contained, and there is no connection between the chapters or a logical structuring principle behind the organization of the book. A better way to structure it would be to first address the impact on major concepts and pathways and then to discuss the specific diseases. The editors have decided not to include some disorders that would be expected in a book about aging-related diseases. For instance, they did not address the intersection of aging and cardiovascular disease with a potential effect on heart disease. On the other hand, some chapters deal with subcellular pathways and detailed molecular mechanisms, which are a more suitable topic for the first volume and are partly described there as well.

**Related Reading:** The textbook should be complemented with Biochemistry and Cell Biology of Aging: Part I, Biomedical Science (Harris JR, Korolchuk VI, eds.).

**Commentary:** Aging is a complex and challenging topic that requires a comprehensive approach covering all aspects of its involvement in different diseases. Aging-related diseases can be successfully treated only if we understand the biochemistry and cell biology of aging. The editors have done their best to address different aspects of age-related changes. However, they failed to cover all clinically relevant conditions associated with aging.

The Foreword of the book states that it has “never been more important or timely for new volumes on the science of ageing” to be published. Given the aging population, this is indeed true, and such editions are urgently needed.

